# MANTA2, update of the Mongo database for the analysis of transcription factor binding site alterations

**DOI:** 10.1038/sdata.2018.141

**Published:** 2018-07-24

**Authors:** Oriol Fornes, Marius Gheorghe, Phillip A. Richmond, David J. Arenillas, Wyeth W. Wasserman, Anthony Mathelier

**Affiliations:** 1 Centre for Molecular Medicine and Therapeutics, Department of Medical Genetics, BC Children’s Hospital Research Institute, University of British Columbia, 950 W 28th Ave, Vancouver, BC V5Z 4H4, Canada; 2 Centre for Molecular Medicine Norway (NCMM), Nordic EMBL Partnership, University of Oslo, 0318 Oslo, Norway; 3 Department of Cancer Genetics, Institute for Cancer Research, Oslo University Hospital Radiumhospitalet, 0310 Oslo, Norway

**Keywords:** Genetic databases, Genetics research, Medical genomics

## Abstract

Interpreting the functional impact of noncoding variants is an ongoing challenge in the field of genome analysis. With most noncoding variants associated with complex traits and disease residing in regulatory regions, altered transcription factor (TF) binding has been proposed as a mechanism of action. It is therefore imperative to develop methods that predict the impact of noncoding variants at TF binding sites (TFBSs). Here, we describe the update of our MANTA database that stores: 1) TFBS predictions in the human genome, and 2) the potential impact on TF binding for all possible single nucleotide variants (SNVs) at these TFBSs. TFBSs were predicted by combining experimental ChIP-seq data from ReMap and computational position weight matrices (PWMs) derived from JASPAR. Impact of SNVs at these TFBSs was assessed by means of PWM scores computed on the alternate alleles. The updated database, MANTA2, provides the scientific community with a critical map of TFBSs and SNV impact scores to improve the interpretation of noncoding variants in the human genome.

## Background & Summary

Understanding the relationship between DNA sequence variation (genotype) and observable traits and diseases (phenotype) is one of the central paradigms of the post-genomics era. While most analyses have focused on the ~2% of the genome that codes for proteins, genome-wide association studies have shown that up to 88% of disease- and trait-associated variants are located in the 98% of the genome that is noncoding^1^. Several computational tools, such as SIFT^2^ and Polyphen^3^, are well established for the assessment of the deleterious impact of coding variation on protein functions yet interpreting the functional impact of noncoding variants continues to be challenging^4^.

Recently, bioinformatics methods have been developed for scoring the impact of noncoding variants based on their pathogenicity and regulatory capacity (Table 1). These methods vary both in their algorithmic approaches and the underlying genomic features used. For instance, evolutionary conservation^5^ can be used to evaluate nucleotides under purifying selection, and experimental data such as histone modifications^6^, chromatin accessibility^7,8^, and DNA methylation^9^ are used to identify biochemically active DNA, which is indicative of regulatory capacity.

Transcription factors (TFs) are sequence-specific DNA-binding proteins that regulate gene transcription^10^. Genomic locations at which TFs interact with DNA are defined as TF binding sites (TFBSs). They are typically short (6–10 bp) and often exhibit degeneracy. Chromatin immunoprecipitation combined with sequencing (ChIP-seq)^11^ provides *in vivo* TF-DNA interactions at ~200–300 bp resolution. These ChIP-seq regions are expected to encompass the 6–10 bp fragments corresponding to TF-DNA interactions (TFBSs). The ReMap database^12^ is a publicly available resource providing an atlas of such regions obtained from 2,829 uniformly processed human ChIP-seq data sets.

The DNA sequences bound by a given TF can be represented as position frequency matrices (PFMs), which count the number of occurrences of each nucleotide within the TFBSs for that TF^13^. PFMs can be converted into probabilistic computational models, namely position weight matrices (PWMs), which can be used to predict TFBSs on any DNA sequence (reviewed by Wasserman and Sandelin^14^). Several databases of PFMs exist^15^, including the recently updated JASPAR database^16^, which stores manually-curated and non-redundant DNA-binding profiles such as PFMs for TFs across six taxonomic groups.

**Figure 1:**
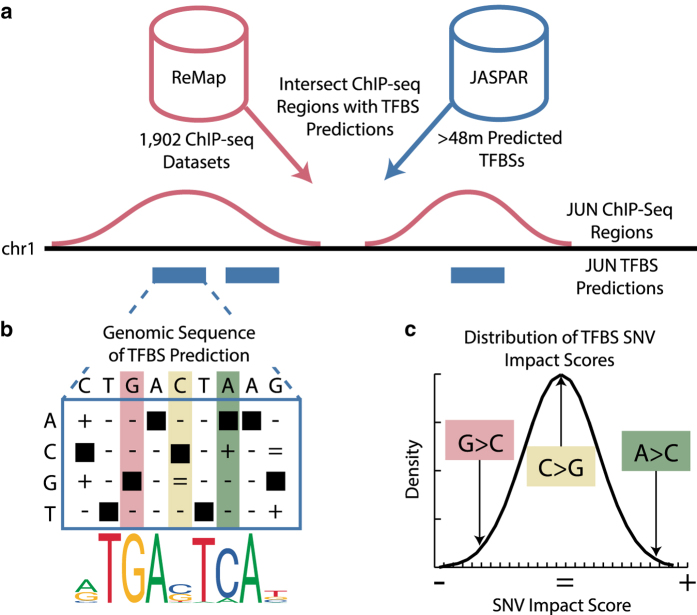
Overview of MANTA2. **a**) Intersection of the ReMap ChIP-seq regions with JASPAR TFBS predictions to produce a set of TFBSs with both experimental and computational evidence of TF binding. A mock example of JUN is given for a region on chromosome one. **b**) A matrix representing the difference in PWM score for all possible SNVs compared to the reference sequence at that TFBS, including negative impact (−), positive impact (+), and no change (0) of score. Black boxes indicate that nucleotides of the reference TFBS sequence are not stored in the database. The sequence logo for JUN is provided below the matrix where the information content is proportional to the size of the nucleotide letters. **c**) Mock distribution of TFBS SNV impact scores when considering all possible SNVs in the TFBS. The distribution is annotated with examples of decreased TF binding capacity (red), no change in TF binding capacity (yellow), and increased TF binding capacity (green).

With most noncoding variants associated with complex traits and disease residing in regulatory sequences^17^, it is expected that some will alter the binding of TFs to DNA^18,19^. Therefore, it is imperative to develop methods that prioritize noncoding variants based on their impact on TF-DNA interactions. In 2015, we developed MANTA, a Mongo database for the analysis of TFBS alterations, to study the impact of regulatory mutations in B-cell lymphomas^20^. The database stores TFBSs in ChIP-seq regions predicted using PWMs derived from the JASPAR database, as well as the potential impact on TF binding of all possible single nucleotide variants (SNVs) that could occur at these TFBSs (Fig. 1). Building on the recent updates of both the JASPAR and ReMap databases, we have largely expanded MANTA. This second release of the database, MANTA2, hosts over 48 million TFBS predictions within ChIP-seq regions of 225 human TFs, covering about 8% of the human genome, together with computed impact scores for all possible overlapping SNVs. Hence, MANTA2 provides the scientific community with a critical map of TFBSs and SNV impact scores for the interpretation of noncoding variants in the human genome.

## Methods

### Transcription factor binding site predictions

From ReMap^12^, we retrieved 1,902 uniformly processed ChIP-seq data sets (*i.e.* sets of ChIP-seq regions) for 227 human TFs for which we had binding profiles in JASPAR^16^. Each ChIP-seq data set was paired with one or more PFMs associated to the ChIP’ed TFs from the JASPAR CORE vertebrates collection (see Supplementary Table 1). For each pair, we intersected the ChIP-seq regions with the corresponding TFBSs predicted for the ChIP’ed TF using bedtools intersect^21^ with "-wa -wb" options to preserve the original coordinates. The PWM-based TFBS predictions are publicly available as part of the JASPAR human genome track at http://expdata.cmmt.ubc.ca/JASPAR/downloads/UCSC_tracks/2018/hg38/tsv/. The intersection resulted in 48,512,399 TFBSs for 225 TFs, covering 255,918,025 bp of the human genome (Fig. 1a). No overlap was found for 2 TFs between the ChIP-seq regions and PWM-based TFBS predictions. Note that all data relates to the build 38 of the Genome Reference Consortium human genome (hg38).

### Computation of SNV impact scores

For each TFBS, we computed the impact on TF binding of all possible overlapping SNVs as described in the manuscript describing MANTA^20^ (Fig. 1b). First, both strands of the 2*n*−1 bp region centered around each possible SNV, where *n* is the length of the considered PWM, were scanned with the corresponding PWM using the TFBS Perl module^22^ (version 0.7.1) to identify the best PWM score on the alternate allele. Note that we only kept the best match per SNV. We then computed the distribution of PWM scores for all these SNVs and calculated the corresponding mean, *m,* and standard deviation, *sd*. For each SNV, the final impact score was calculated as the Z-score of its TFBS score, *S*, within the distribution of alternate PWM scores at that TFBS (*i.e.* (*S*−*m*)/*sd*). Users can refer to the webinar video describing the original MANTA database (http://www.cisreg.ca/Webinars/JASPAR_BioPython_MANTA.flv). Therefore, for each SNV, MANTA stores its associated reference and alternate TFBS PWM scores and locations, along with the computed impact score.

### Validation using heterozygous TF-binding events

**Figure 2:**
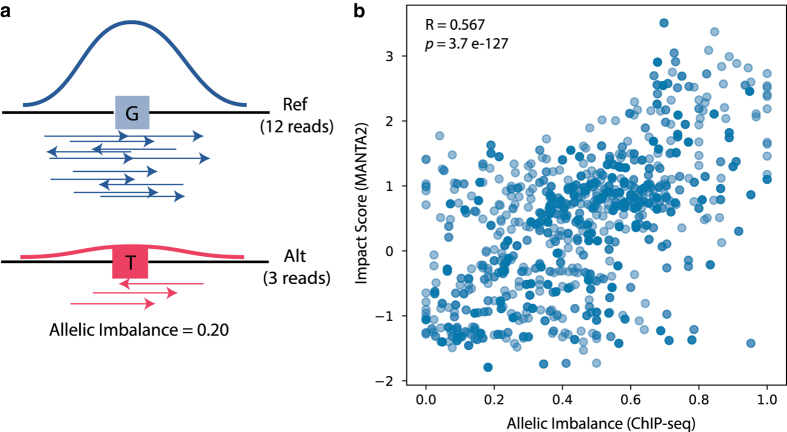
Assessing MANTA2 impact scores with heterozygous TF-binding events. **a**) Allelic imbalance is calculated as the number of ChIP-seq reads mapped on the alternate allele divided by the total number of reads mapped at heterozygous sites. **b**) MANTA2 impact scores correlate with allelic imbalance of ChIP-seq data. Events (blue dots) are plotted with respect to their allelic imbalance of ChIP-seq reads (*x*-axis) and impact scores from MANTA2 (*y*-axis). The Pearson coefficient (R) and *P*-value (*p*) of the correlation between allelic imbalance and impact score are provided in the plot.

We downloaded ChIP-seq data for 35,703 TF-binding events at heterozygous sites in GM12878 and HeLa cells for 36 different TFs^18^. For each event, allelic imbalance was calculated as the number of ChIP-seq reads mapped on the alternate allele divided by the total number of reads mapped at that position (Fig. 2a). The coordinates from the original publication refer to the hg19 version of the human genome; we used the liftOver tool from the UCSC Genome Browser^23^ to convert them to the hg38 assembly (the conversion process failed for 12 coordinates).

### Code availability

MANTA2 is freely distributed as a GitHub repository at https://github.com/wassermanlab/MANTA2.

## Data Records

The Mongo database dump of MANTA2, is deposited as a tarball on Zenodo (Data Citation 1).

## Technical Validation

The quality and technical validation of the ChIP-seq data and TFBS predictions is described in the 2018 manuscripts of ReMap^12^ and JASPAR^16^, respectively, and is summarised below.

### ReMap ChIP-seq data

ReMap ChIP-seq datasets were uniformly processed using a well-established pipeline^12^. ChIP-seq reads were aligned to the human genome using bowtie2 (ref. 24) (version 2.2.9) using options “-end-to-end” and “-sensitive”. When necessary, reads were trimmed and polymerase chain reaction duplicates were removed from the alignments with samtools rmdup^25^. ChIP-seq regions were identified using the MACS2 peak-calling tool^26^ (version 2.1.1.2) with default parameters. The quality of all ChIP-seq datasets was assessed based on metrics developed by the ENCODE consortium^27^.

### JASPAR TFBS predictions

JASPAR TFBSs were predicted by scanning the human genome using two different methods^16^: the TFBS Perl module^22^ (version 0.7.1) and FIMO^28^, as distributed within the MEME suite^29^ (version 4.11.2). FIMO is one of the best performing tools for scanning DNA sequences with PWMs to predict TFBSs^30^. To scan the human genome with the BioPerl TFBS module, PFMs were converted to PWMs and predictions with a relative score ≥0.8 were kept. In preparation for the FIMO scan, PFMs were reformatted to MEME motifs and motifs that matched with a *P*-value <0.05 were kept. For quality control, TFBS predictions that were not consistent between the two methods were filtered out. Such consistency ensures, for instance, technical validation for the coordinates of the TFBS predictions.

### MANTA2

The technical validation of MANTA2 involved assessing data quality and database integrity controls. A spot check data quality control was performed using the UCSC Genome Browser^23^. For 15 randomly selected TFBSs (of different TFs) from MANTA2 we manually checked that: 1) the TFBS overlapped a ReMap ChIP-seq region associated with that TF; 2) the JASPAR PFM matched the start, end, and strand stored for that TFBSs; and 3) the stored SNVs for that TFBS had the expected impact on TF binding. Moreover, we assessed the usefulness of MANTA2 impact scores on an external dataset of heterozygous TF-binding events^18^. As expected, the allelic imbalance calculated for ChIP-seq reads (see Methods) significantly correlated with the impact scores from MANTA2 (Pearson correlation coefficient=0.567, *P*-value=3.7e-127; Fig. 2b). Additionally, we checked the database integrity for MANTA2 by dumping and restoring the database on common operating systems and workstations. Finally, we tested the command line and web interface access to MANTA2 (see Usage Notes section) to interpret variant files in VCF, GFF, and BED format.

## Usage Notes

MANTA2 can be accessed either programmatically or via its web interface. To access the database programmatically, users must first clone (*i.e.* “git clone https://github.com/wassermanlab/MANTA2.git”) or download MANTA2 from GitHub (see Code availability in the Methods section). The script “search_manta2.py” provides programmatic access to MANTA2. It requires the following inputs:

- The name of the MANTA2 database in the MongoDB system (option “-d”)
- The name of the server where the MongoDB system is hosted (option “-H”)
- A user with “read” privileges to the MANTA2 database (option “-u”)
- The password for that user (option “-p”)
- A file containing a list of variants in “VCF”, “BED” or “GFF” format (option “-i”)

Non-mandatory options include:

- The format of the input variant file (option “-t”; by default the script tries to identify the input format automatically)
- The name of a file to output the results (option “-o”; by default is set to the standard output stream (stdout))

As a usage example, the MANTA2 database hosted by the Wasserman lab can be accessed as follows: “python search_manta2.py -d manta2 -H manta.cmmt.ubc.ca -u manta_r -p mantapw -i <variant file>”.

A variant file can be obtained by executing the shell script: “bash ./examples/get_VCF_example.sh”.

The resulting VCF file (*i.e.* “chr20.vcf”) contains high-confidence SNP, small indel, and homozygous reference calls on chromosome 20 from the Genome in a Bottle (version 3.3.2) sample HG001 (ref. 31). In response, “search_manta2.py” returns all TFBS predictions potentially impacted by these variants as tab-separated values. For each TFBS alteration, the script provides the variant information along with the associated wild-type (reference) and mutated (alternative) TFBS information, including:

- the chromosome and position of the variant;
- the reference and alternative alleles at that genomic location;
- the mutation ID (if the input file format allowed for it, otherwise the field is displayed as “.”);
- the TF name and associated JASPAR profile ID;
- the start, end and strand, as well as the absolute (raw) and relative scores for both the reference and alternative TFBSs;
- and the impact score.

Users who plan on performing large numbers of searches should create a local build of the MANTA2 database. Instructions are provided in the “README.md” file of the GitHub repository.

The MANTA2 database hosted by the Wasserman lab can also be accessed via a dedicated web server at http://manta.cmmt.ubc.ca/manta2. Similar to the “search_manta2.py” script, the server requires as input a list of variants in VCF, GFF, or BED format (see help page), and it returns all TFBS predictions potentially impacted by these variants as a tab-separated values table. The table can be sorted on any column by clicking on the column header.

## Additional information

**How to cite this article**: Fornes, O. *et al*. MANTA2, update of the Mongo database for the analysis of transcription factor binding site alterations. *Sci. Data* 5:180141 doi: 10.1038/sdata.2018.141 (2018).

**Publisher’s note**: Springer Nature remains neutral with regard to jurisdictional claims in published maps and institutional affiliations.

## Supplementary Material



Supplementary Table 1

## Figures and Tables

**Table 1:** List of published tools with the capacity to evaluate the impact of noncoding variants.

**Method**	**Designed for**	**Algorithmic approach**	**Genomic features**	**PMID**
CADD	pathogenicity	support vector machine	conservation, epigenomic annotations	24487276
CpGenie	impact on methylation	deep neural network	conservation, epigenomic annotations, TFBS alterations	28334830
DANN	pathogenicity	deep neural network	conservation, epigenomic annotations	25338716
DeepSEA	regulatory potential	deep neural network, logistic regression classifier	conservation, epigenomic annotations, TFBS alterations	26301843
deltaSVM	regulatory potential	support vector machine	epigenomic annotations, TFBS alterations	26075791
Eigen	pathogenicity	spectral clustering	conservation, epigenomic annotations	26727659
FATHMM	pathogenicity	hidden Markov model	conservation, epigenomic annotations	28968714
fitCons	fitness consequence	generative probability, genome partitioning	conservation, epigenomic annotations	25599402
FunSeq2	cancer pathogenicity	feature-based scoring, PWM scoring, somatic hotspots	conservation, epigenomic annotations, TFBS alterations	25273974
GWAVA	pathogenicity	random forest	conservation, epigenomic annotations, TFBS alterations	24487584
LINSIGHT	regulatory potential	linear regression, generative probability	conservation, epigenomic annotations, TFBS alterations	28288115
MANTA	regulatory potential	PWM scoring	TFBS alterations	25903198
RegulomeDB	regulatory potential	feature-based scoring, PWM scoring	conservation, epigenomic annotations, TFBS alterations	22955989
ReMM	pathogenicity	random forest	conservation, epigenomic annotations	27569544
RVSP	regulatory potential	random forest	conservation, epigenomic annotations	27406314
SNP2TFBS	regulatory potential	PWM scoring	TFBS alterations	27899579
For each “Method”, we describe its “Intended use”, “Algorithmic approach”, underlying “Genomic features” and PubMed ID (“PMID”) of the corresponding publication.				
